# Discharge Plasma Treatment as an Efficient Tool for Improved Poly(lactide) Adhesive–Wood Interactions

**DOI:** 10.3390/ma14133672

**Published:** 2021-06-30

**Authors:** Mariusz Ł. Mamiński, Igor Novák, Matej Mičušík, Artur Małolepszy, Renata Toczyłowska-Mamińska

**Affiliations:** 1 Institute of Wood Sciences and Furniture, Warsaw University of Life Sciences—SGGW, 159 Nowoursynowska St., 02-776 Warsaw, Poland; 2 Polymer Institute, Slovak Academy of Sciences, 84236 Bratislava, Slovakia; igor.novak@savba.sk (I.N.); matej.micusik@savba.sk (M.M.); 3 Faculty of Chemical and Process Engineering, Warsaw University of Technology, 1 Waryńskiego St., 00-645 Warsaw, Poland; artur.malolepszy@pw.edu.pl; 4 Institute of Biology, Warsaw University of Life Sciences—SGGW, 159 Nowoursynowska St., 02-776 Warsaw, Poland; renata_toczylowska@sggw.edu.pl

**Keywords:** polylactide, plasma, treatment, wood bonding

## Abstract

Poly(lactide) (PLA) films obtained by thermoforming or solution-casting were modified by diffuse coplanar surface barrier discharge plasma (300 W and 60 s). PLA films were used as hot-melt adhesive in joints in oak wood. It was demonstrated that lap shear strength increased from 3.4 to 8.2 MPa, respectively, for the untreated and plasma-treated series. Pull-off tests performed on particleboard for the untreated and treated PLA films showed 100% cohesive failure. Pull-off strength tests on solid oak demonstrated adhesion enhancement from 3.3 MPa with the adhesion failure mode to 6.6 MPa with the cohesion failure mode for untreated and treated PLA. XPS revealed that carbonyl oxygen content increased by two-to-three-fold, which was confirmed in the Fourier-transform infrared spectroscopy experiments of the treated PLA. The water contact angle decreased from 66.4° for the pristine PLA to 49.8° after treatment. Subsequently, the surface free energy increased from 47.9 to 61.05 mJ/m^2^. Thus, it was clearly proven that discharge air plasma can be an efficient tool to change surface properties and to strengthen adhesive interactions between PLA and woody substrates.

## 1. Introduction

Polylactide (PLA) is one of the most popular green polymers, as its market value in 2019 reached 535.6 million USD [1]. PLA is fully biodegradable, and it can also be obtained synthetically from lactic acid or biotechnologically from the bacterial fermentation of starch [2,3]. Because PLA is biodegradable and may be obtained from renewable resources through fermentation, the CO_2_-neutral life cycle of PLA may be established [4]. As the mechanical and physical properties of PLA are often comparable to those of polystyrene, it has become one of the most used materials for bioplastics production, though with relatively higher costs in comparison to conventional plastics [5]. In addition to bioplastics, PLA has found applications in the production of fibers, coatings, films, and foams [6]. It has also attracted great attention as a biomedical material because of its biocompatibility, bioresorbability, and non-toxic degradation, which allows it to be applied for the production of medical implants, sutures, prostheses, vascular stents, and tissue engineering [7]. Pure PLA is a thermoplastic material with typical mold temperature of 120–210 °C, but its main drawbacks are poor ductility, poor toughness, low thermal stability, and high flammability, all of which limit its applications, e.g., in electronics or construction materials [8]. The poor thermal stability of PLA is especially important in the case of its application in hot-melt adhesives, where the material needs to be stable during the application process. The improvement of PLA’s properties has been realized through pre-drying, peroxide modification, plasticizing (with, e.g., poly (ethylene glycol)), copolymerizing, and blending with poly (vinyl acetate), poly (ethylene oxide) [9,10,11]. For example, a copolymer of PLA and poly(ε-caprolactone) was used as a hot-melt adhesive for the industrial lamination of cardboard and PLA-based films [12]. It was observed that despite its poor surface strength, such an adhesive was sufficient for industrial applications where it should degrade at the same time as its package. PLA hydrophilicity was also improved through controlled depolymerization to obtain PLA esters of lower molecular weights [13].

PLA is incompatible with hydrophilic materials such as wood [2]. The observed poor mechanical properties of wood–PLA composites are caused by the poor compatibility of hydrophilic wood and hydrophobic PLA, which results in poor interfacial adhesion. Usually in wood/polymer composites, adhesion is improved by chemical methods with the use of sodium hydroxide, polyolefin, isocyanates, triazines, maleimides, polyols, polyethylene glycol, or mineral fillers [14,15,16,17,18]. The application of these compounds allows for the enhancement of the toughness and strength of PLA–wood composites, but they have a complicated production process and increased costs. At the same time, the most important is the fact that the chemical modification of PLA introduces chemicals and additives to the biodegradable material that eliminate chemically modified PLA–wood composites from the group of environmentally benign materials. The best ways to improve PLA–wood adhesion from the environmental point of view are physical methods like plasma treatment, which is a surface modification method commonly used to increase the surface free energy and adhesion of polymeric materials [19]. Plasma modification has been successfully used to enhance the performance of wood–polypropylene composites with an efficiency comparable to chemical methods, where an increase of over 100% was obtained in tensile modulus in comparison to untreated polypropylene after plasma treatment. Surface plasma treatment was also successfully used to improve paint adhesion to wood (approximately 50% decrease in water contact angle and a two-fold increase in the surface free energy of wood after plasma treatment in comparison to untreated samples), which was connected to the generation of large amount of oxygen-containing functional groups [20]. A report by Sauerbier and co-workers confirmed increased surface free energy after plasma treatments and greatly improved adhesion [21]. The plasma treatment of PLA was also used to enhance PLA degradation by increasing its hydrophilicity [22]. In this case, the plasma-induced formation of hydroxyl end-groups allowed for the enhancement of PLA hydrolytic degradation.

Usually, PLA is used in copolymers for adhesive purposes. Here, we propose the application of pure plasma-treated PLA as an adhesive, without any chemical modification that would influence its biodegradability or reduce environmental benignancy.

The aim of this work was to demonstrate the plasma modification of PLA at ambient pressure as an efficient tool of practical significance to improve adhesive interactions between PLA and solid wood, as well as investigate the chemical changes in the PLA surface induced by the treatment.

## 2. Materials and Methods

### 2.1. PLA Film Preparation

A commercially available PLA polymer (L95-M, Sultzer, Winterthur, Switzerland) with a Melt Flow Index (2.16 kg/190 °C) of 22–23 g/10 min, 5% D-isomer, a melting point of 153 °C, and a crystallinity of 20–25% was used in experiments [23]. Two series of 0.1-mm thick films were produced: (1) PLA-T was thermoformed from pellets by flat pressing at 200 °C, and (2) PLA-MC was cast from solution in methylene chloride and left for 7 days to evaporate (20 °C; 65% relative humidity).

### 2.2. Barrier Discharge Plasma Wood Treatment

**Figure 1:**
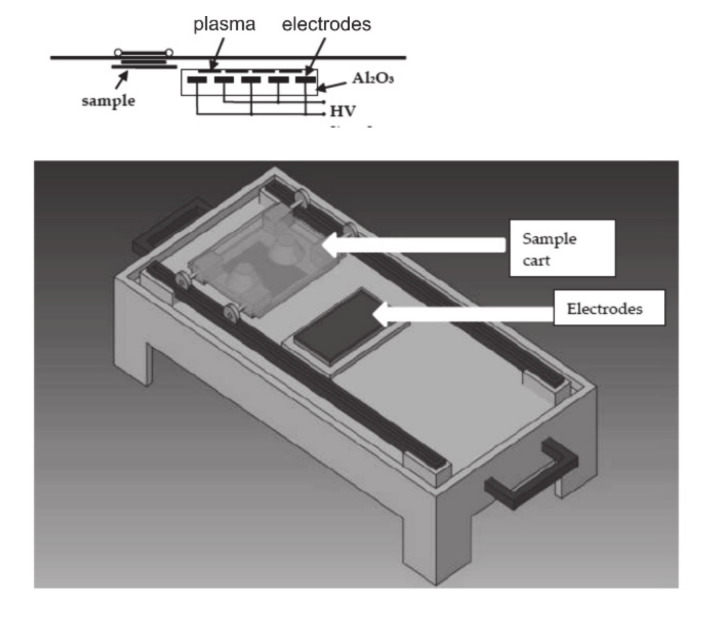
Scheme of DCSBD plasma set up.

In this work, a diffuse coplanar surface barrier discharge (DCSBD) plasma source (Masaryk University, Brno, Czech Republic) in air under atmospheric pressure was used to modify the surface of the PLA film. The DCSBD plasma source was operated at 300 W. The DCSBD plasma set up is schematically shown in Figure 1.

Two parallel-banded systems of electrodes made of Ag paste effectively generated the cold plasma. The electrodes were placed in 96% Al_2_O_3_, which protected the electrodes from direct contact with the plasma and prolonged their lifetime. The plasma console was also connected to a cooling system to prevent overheating. A high-frequency sinusoidal voltage (~15 kHz, with a Um of ~10 kV) was applied to the electrodes, leading to macroscopically homogenous plasma generation and uniform surface treatment. PLA film specimens of 25 × 60 mm^2^ were treated for 60 s at atmospheric pressure in air.

### 2.3. Fourier-Transform Infrared Spectroscopy (FTIR)

FT-IR spectra were collected using a Nicolet iS10 spectrometer (Thermo Fisher Scientific, Waltham, MA, USA) in the attenuated total reflectance (ATR) mode on a diamond crystal. The measurements were performed in the range of 400–4000 cm^−1^. The samples were measured before and after plasma treatment.

### 2.4. X-Ray Photoelectron Spectroscopy (XPS)

XPS signals were recorded using a Thermo Scientific K-Alpha XPS system (Thermo Fisher Scientific, Loughborough, UK) equipped with a micro-focused monochromatic Al Ka X-ray source (1486.68 eV). The Thermo Scientific Avantage software, version 5.9921 (Thermo Fisher Scientific), was used for digital acquisition and data processing. Spectral calibration was determined by using the automated calibration routine and the internal Au, Ag, and Cu standards supplied with the K-Alpha system.

### 2.5. Oak Wood Bonding and Shear Strength Testing

The shear strengths of the adhesive joints (R_t_) between oak wood slabs bonded with PLA as a hot-melt adhesive were measured by the tensile testing of the single overlapped adhesive joints. The joints were prepared using oak slabs with dimensions of 60 (length) × 20 (width) × 2 mm (thickness), as well as lap dimensions of 20 × 20 mm^2^. The wood slabs were bonded together in the hydraulic hot press (Fontijne, The Netherlands) with PLA film as a hot-melt adhesive at the temperature of 160 °C for 60 s under a pressure of 0.8 MPa, and then they were cooled down in a cold press for 5 min. The adhesive joints were subsequently tested using a 5 kN Instron 4301 universal testing machine (Instron Corp., Norwood, MA, USA) at a constant crosshead speed of 10 mm/min. The experiments were performed as described elsewhere [24]. Shear strength (R_t_) was calculated using Equation (1):

R_t_ = F_max_/S (1)

where F_max_ is the maximum force in N and S is lap area in mm^2^.

### 2.6. Pull-Off Testing

**Figure 2:**
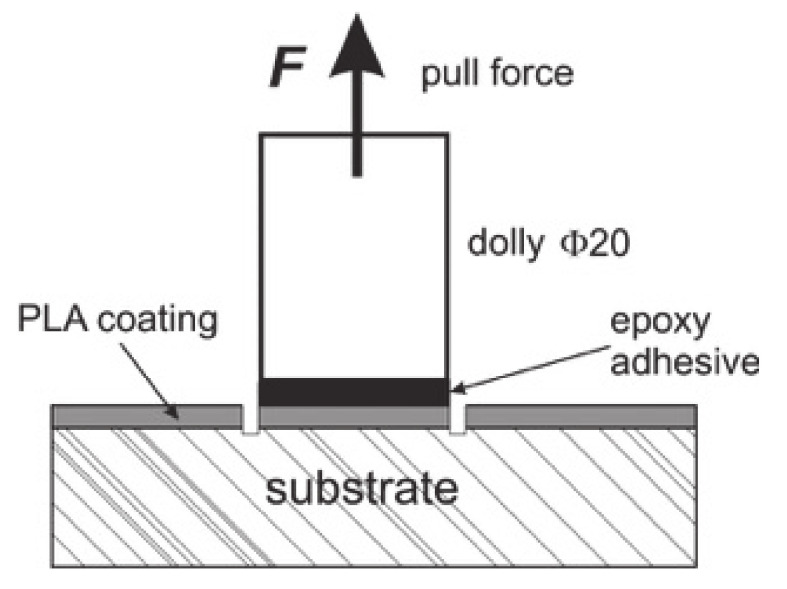
Pull-off specimen.

Pull-off tests were performed on particleboard (16 mm and 850 kg/m^3^) and on solid oak wood. Both substrates were coated with PLA films in a hot press (200 °C, 0.8 MPa, and 45 s). A Positest^®^AT-A pull-off adhesion tester (DeFelsko Corp., Ogdensburg, NY, USA) equipped with 20-mm dollies was used in testing at a pull rate of 0.2 MPa/s. The pull-off specimen structure is shown in Figure 2.

### 2.7. Contact Angle Measurements and Surface Free Energy (SFE) Calculations

Contact angle measurements were done on a Phoenix 300 contact angle analyzer (Surface Electro Optics, Suwon City, Korea). Distilled water and diiodomethane were used as the reference liquids. The contact angle was read 20 s after droplet deposition. The Owens-Wendt method was used in the calculations of surface free energy [25]. The measurements were performed immediately after plasma treatment since it has been proven that wetting is reduced with time [26].

### 2.8. SEM Analysis

Micrographs were made on a Hitachi s5500 scanning electron microscope (Hitachi High-Technologies Co., Ltd., Tokyo, Japan) at 10,000 × magnification. Images were taken at a voltage of 1 kV without gold sputtering in the LA-BSE (mass contrast) mode.

## 3. Results and Discussion

Electric discharge in low-temperature plasma is a source of highly reactive transient species that affect the chemical structure of a material [27]. These species are excited atoms, ions, or free radicals that result in the scission of existing bonds and formation of new functional groups. The changes affect surface hydrophilic/hydrophobic character and change the surface free energy (SFE) of a material [26,28].

### 3.1. Chemical and Physical Changes in PLA Surface

**Figure 3:**
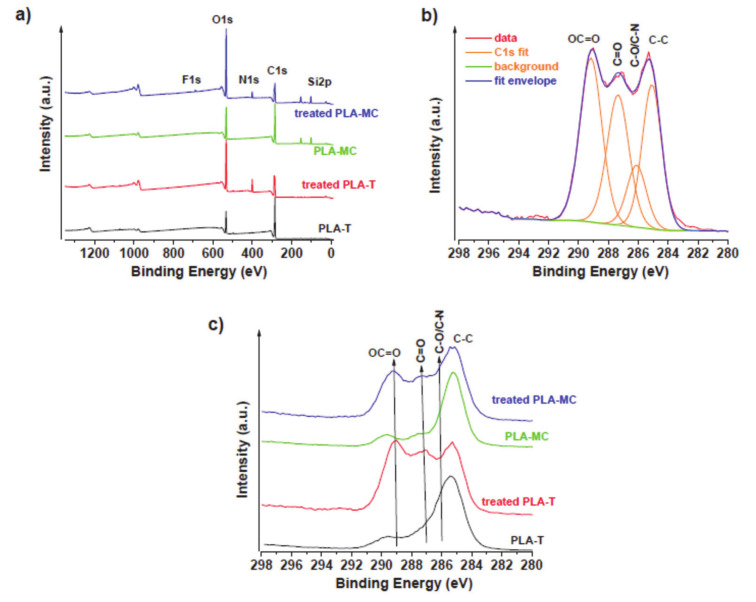
XPS of (**a**) survey of all samples, (**b**) C1s deconvolution of treated PLA-T, and (**c**) C1s region of all samples.

**Figure 4:**
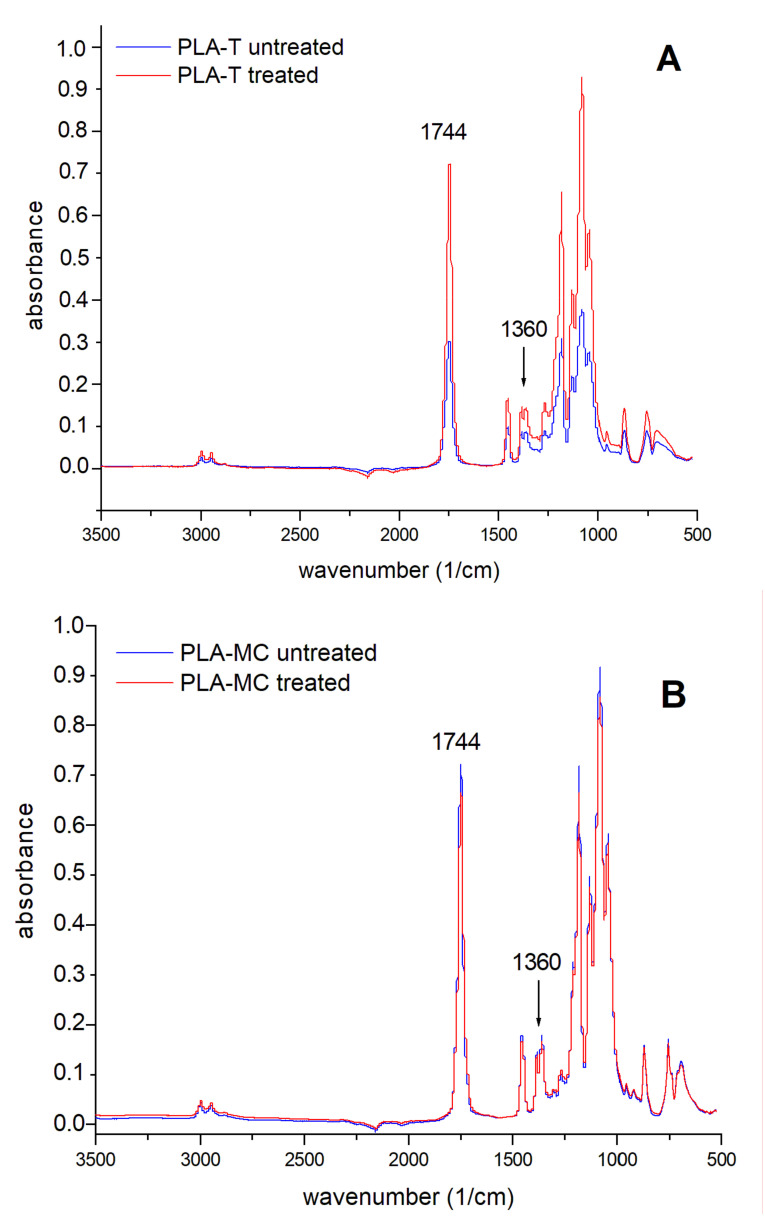
FTIR spectra: (**A**) thermoformed film (PLA-T) and (**B**) solution cast film (PLA-MC).

In order to determine the changes in chemical structure of PLA surface modified by atmospheric plasma in air, XPS (Table 1 and Figure 3) and FTIR spectra (Figure 4) were recorded. In general, the higher the content of oxidized functional groups, the stronger the interfacial adhesive interactions.

#### 3.1.1. X-ray Photoelectron Spectroscopy

The C1s peaks in the XPS spectrum were found to be associated with carbonyl, carboxyl, and alkoxy groups. As shown in Table 1 and Figure 3, a significant decrease in the C1s C–C component centered at ~285 eV and increases in the C1s C=O (at ~287 eV) and O-C=O (at ~289 eV) components revealed that atmospheric plasma treatment in air resulted in a two-to-three-fold increase in oxygen-rich functional groups on the surface (Figure 3). If we compare the concentration in the peak C1s of the carbonyl C=O groups in the PLA-T (thermoformed sample) after modification with atmospheric plasma in air, it can be stated that it had a substantial increase from 14.8 to 27.3 at. %. For PLA-MC (cast sample), an even more significant increase in the concentration of C=O groups from 9.7 to 23.2 at. % was observed after plasma modification. A more significant increase than for the C=O groups was observed in the C1s peak for the O–C=O ester groups, whose content for PLA-T and PLA-MC after plasma treatment increased from 10.1 to 34.4 at. % and from 11.1 to 27.8 at. %, respectively.

As shown in Table 1, both samples (PLA-T and PLA-MC) had some contamination, such as phosphate (P2p at ~134 eV) and sulphate (S2p at 168 eV), on their surface. The PLA-T sample also showed traces of NaCl (Na1s at ~1071 eV and Cl2p at ~198 eV) and some silanes (Si2p at ~102 eV). PLA-MC showed a high amount of silane (9.0 at. % of Si-O and 48.0 at. % of C-C) contamination, probably resulting from the casting method. As a consequence of this contamination, the plasma treatment was less effective and resulted in lower carbonyl (C=O) and carboxyl (OC=O) contents in the case of treated PLA-MC. In both cases, there was clear increase of C-N (N1s at ~400 eV) coming from the high reactivity of the surface with ambient air. Thus, it seems that hot-pressing is a more reliable method for film-forming and provides higher purity of films.

#### 3.1.2. SEM Analysis

**Figure 5:**
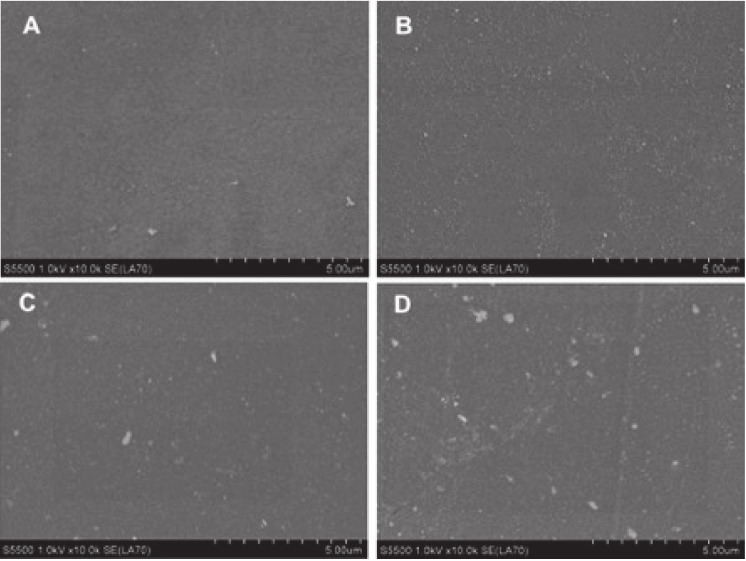
SEM images of PLA: (**A**) untreated PLA-T; (**B**) plasma-treated PLA-T; (**C**) untreated PLA-MC; (**D**) plasma-treated PLA-MC.

In order to explain whether physical changes in the PLA surface were induced by plasma treatment, SEM analysis was performed. As Figure 5 shows, the microstructure of the surface was not affected. No changes in surface geometry could be seen at 10 k magnification. Regardless of the PLA film preparation, the surface was compact and smooth, in accordance with the observations of others [29]. However, it should be noted that 60-s modification time was not sufficient to affect surface geometry. As was reported by Moraczewski et al., longer plasma treatment times (5–30 min) are required for cracks and cavities to occur [30].

On the other hand, the bright spots in the SEM images shown in Figure 4A–D indicate the inclusion of other element impurities in the entire polymer volume, both before and after treatment. This finding corresponds to the XPS results, which revealed the presence of silica, phosphorus, and sulfur (Table 1). The observed impurities were quite evenly dispersed and present both in pristine and modified samples, so it is very likely that they neither improved nor hampered the adhesive interactions between the PLA film and the substrates.

#### 3.1.3. Fourier-Transform Infrared Spectroscopy

The FTIR spectra shown in Figure 4 confirmed the findings of the XPS experiment, though no new bands occurred. Typical characteristic PLA peaks were present: 2994–2944 cm^−1^ (C–H), 1744 cm^−1^ (C=O), 1450 cm^−1^, and 1360 cm^−1^ (–CH_3_), as well as O–C=O stretching at 1185 cm^−1^ [31,32]. In order to display the changes in the abundancy of carbonyl groups, the relative intensities of the C=O/C–H bands present at 1744 and 1360 cm^−1^, respectively, were compared. Thus, in the untreated PLA-T spectrum, the 1744/1360 cm^−1^ ratio was 3.47, while in the treated PLA-T spectrum, the ratio increased to 5.01; this indicated an increase in carbonyl group abundancy associated with a decrease in the number of methyl groups. Unfortunately, the above-mentioned 1744/1360 cm^−1^ ratios in the PLA-MC film cast from methylene chloride were 4.02 and 4.05 for the untreated and treated samples, respectively. Thus, this finding did not prove the apparent increase in C=O content. However, such discrepancies between XPS and FTIR results have already been reported in the literature, where only few changes in the 1000–1250 cm^−1^ (C–C/H) and 1750 cm^−1^ (C=O) regions have been found [21].

### 3.2. Contact Angle and Surface Free Energy (SFE)

It is known from the adsorption theory of adhesion that the relationship between the SFEs of the substrate and the adhesive affects adhesive interfacial forces, and their magnitude strongly depends on the surface properties of both phases [33]. Plasma modification can effectively change the chemistry of the surface layer of polymeric materials, increase their polar character, increase SFE, and subsequently improve their bondability. As the methyl groups in PLA macromolecules contribute to their hydrophobic character, their oxidation or grafting with an oxidized moiety can provide the polymer surface with better wetting by polar liquids. The approach allows one to obtain greatly improved adhesion, even for such low energy substrates like polyolefins or PTFE that lack functional groups [34].

PLA is not considered a rather high energy material, as the reported SFEs of pristine PLA have ranged between 40 and 86 mJ/m^2^ depending on the grade, manufacturer, and processing factors [29,35,36].

In our experiments, SFE calculations were based on the contact angles determined for water and diiodomethane as the wetting liquids. From the results shown in Table 2, it is clear that plasma treatment improved water wetting, as the contact angle decreased from 66.3° and 61.4° for the untreated PLA-T and PLA-MC, respectively, to 49.7° and 57.4° for the treated films (Supplementary Materials Figure S1). The results were especially apparent for the thermoformed PLA-T film and less obvious for the methylene chloride solution cast PLA-MC film.

However, these results remained consistent with the findings from XPS and FTIR that showed the formation of new oxygen-bearing functional groups. The presented contact angles for both the pristine and plasma-treated PLA-T and PLA-MC corresponded to those reported in the literature [36,37,38], as did the observed increase in SFE from 47.9 and 45.8 mJ/m^2^, respectively, to 61.0 and 61.2 mJ/m^2^ [30,39]. It must be also noticed that changes induced by plasma are prone to disappear with time [26,37].

### 3.3. Effect of Plasma Treatment on the Performance of Adhesive Joints

Polyester films are used in the lamination of particleboards and fiberboards in furniture industry [38]. PLA films seem to be an option for this application. In order to determine the effect of plasma treatment on interfacial interactions, mechanical tests of adhesive joints between PLA and woody materials were performed. The first experiment involved tensile shear strength in solid wood lap specimens bonded with PLA as a hot melt. The observed shear strengths are shown in Table 2. It is clear that the improved mechanical properties of the joints are associated with increased SFE after plasma treatment. The gains in strengths reached 240% and 160%, respectively, for the PLA-T and PLA-MC films. Comparable gains for plasma-treated PLA bonded to wood with a PVAc adhesive (0.55–3.72 MPa) were reported by Kariž at al. [40]. On the other hand, Mandolfino et al. [41] observed increases of between 52.5% and 386.6% in lap-shear tests performed on a plasma-treated polypropylene film–epoxy adhesive. 

**Figure 6:**
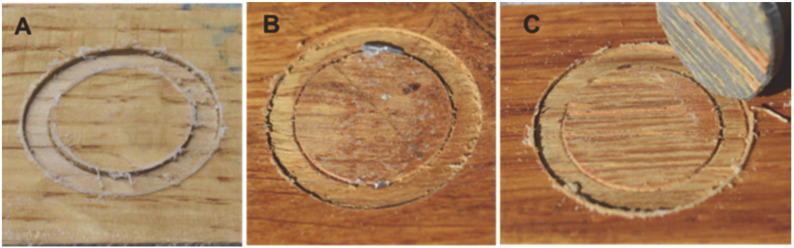
Failure locus after pull-off tests on solid oak wood: (**A**) adhesive failure mode on the untreated PLA/epoxy adhesive interface, (**B**) adhesive failure mode on the treated PLA/wood interface, and (**C**) mixed adhesive/cohesive failure mode on the treated PLA/wood interface.

**Figure 7:**
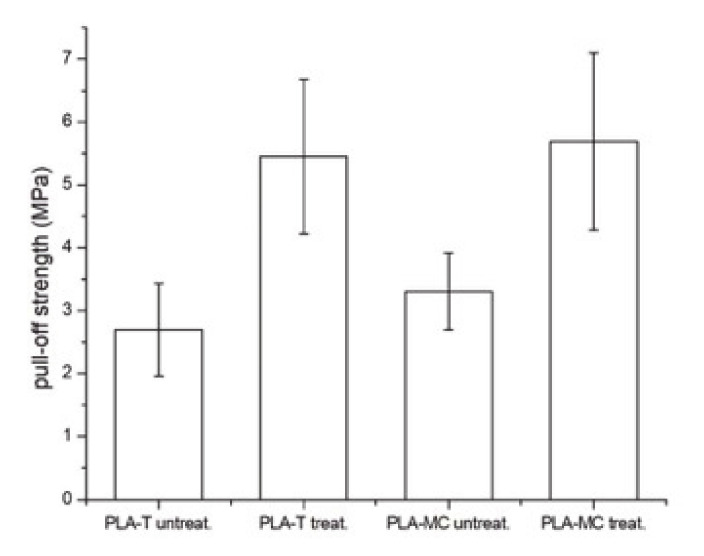
Pull-off test results of the bondline in PLA-laminated oak wood.

The other experiment, the pull-off test, is commonly used in the examination of the joint between laminates and substrates [42]. A pull-off test in a particleboard laminated with PLA films without an additional adhesive (Figure 2) revealed that bondline strengths exceeded the minimum surface soundness of the particleboards defined by the EN 312 standard (0.8 MPa) and a 100% cohesive failure in substrate occurred, which proved to allow for sufficient bonding that was comparable to that of the commercial adhesives [42,43]. However, the true strength of the joint was not determined due to the low cohesion in the substrate. In order to resolve that issue, pull-off tests were performed on PLA-laminated oak wood (Figure 6). The results shown in Figure 6 and Figure 7 indicate that untreated PLA-T and PLA-MC exhibited bond strengths of 2.7 ± 0.7 and 3.3 ± 0.6 MPa, respectively, with apparent adhesive failure at the PLA/epoxy interface (Figure 6A), while plasma-treated PLA-T and PLA-MC specimens exhibited strength increases to 5.4 ± 1.2 and 5.7 ± 1.4 MPa, respectively, corresponded to increases of 200% and 173%. In addition, the failure mode at the untreated PLA/epoxy interface (Figure 6A) transformed into the adhesive mode at the treated PLA/wood interface (Figure 6B) and the mixed adhesive/cohesive mode on the treated PLA/wood interface (Figure 6C). These phenomena undoubtedly confirmed the assumptions of a positive effect of plasma treatment on the strengthened interface adhesives interactions resulting from the changed chemistry of the surface proved by XPS and FTIR. The obtained improvements were higher than those from [21], where a 67.6% increase in coating adhesion after plasma treatment was reported, as were improvements better than those found from pull-off tests in thermoplastic polyurethane/beech and spruce veneers—1.0 and 2.5 MPa, respectively [44]. A similar apparent enhancement in bondline performance was reported by Novák and co-workers for plasma-treated polyester films in peel tests (270–325%) [28], as well as by Nečasová and co-workers, who observed significant increases in the cohesive failure mode rate for plasma-treated substrates [45].

The above results confirmed the hypothesis that discharge air plasma treatment could be an efficient tool for increasing the SFE of PLA films by changing the chemistry of their surface and subsequently improving adhesion interactions with polar substrates. Such an approach resulted in increased joint strength and was demonstrated to be applicable in both coating and bonding with PLA films without a significant reduction of the mechanical properties of the joints. However, it is necessary to keep in mind that the final results of the bonding and mechanical performance of bondlines are governed by a wide range of parameters such as substrate surface chemistry and microstructure, process parameters, and experimental conditions [46,47,48].

## 4. Conclusions

It was proven that PLA films, when properly treated by a physical factor such as discharge barrier plasma, can be used as the only hot-melt adhesives for woody materials or applied in the lamination of wood-based composites without additional adhesives. The performance of the PLA-based adhesive joints was comparable to a large extent with that of those based on traditional adhesives. Consequently, such systems are more environmentally benign because they only use biodegradable components. The approach can be used in the design of new eco-friendly market products conforming to the principles of green chemistry and sustainable development.

## Figures and Tables

**Table 1:** Surface chemical composition of PLA-T and PLA-MC series, as determined by XPS.

**Sample**	**Surface Chemical Composition (Atomic %)**		
	C1s C-C/C-O/C = O/OC = O	O1s	Si2p/P2p/S2p/N1s/F1s/Na1s/Cl2p
Untreated PLA-T	79.4 30.4/29.2/11.7/8.0	18.3	0.8/0.3/0.2/0.7/-/0.3
Treated PLA-T	53.5 14.4/6.2/14.6/18.4	35.2	0.2/0.3/0.2/10.1/-/0.2/0.3
Untreated PLA-MC	67.0 48.0/5.1/6.5/7.4	23.3	9.0/0.4/-/-/0.3/-/-
Treated PLA-MC	43.8 14.1/7.3/10.2/12.2	40.3	9.0/0.5/0.1/5.4/0.9/-/-

PLA-T—thermoformed; PLA-MC—solution cast.

**Table 2:** Wetting, SFE, and tensile shear strength (R_t_) of PLA-T and PLA-MC series.

Series	Contact Angle (°)		SFE (mJ/m^2^)	R_t_ (MPa)
	Water	Diiodomethane		
PLA-T untreated	66.3 ± 1.4	42.6 ± 0.7	47.9	3.4 ± 0.4
PLA-T treated	49.7 ± 2.3	27.9 ± 1.5	61.0	8.2 ± 0.7
PLA-MC untreated	61.4 ± 1.5	66.5 ± 1.7	45.8	4.8 ± 0.5
PLA-MC treated	57.4 ± 0.8	35.8 ± 0.7	61.2	7.7 ± 0.6

PLA-T—thermoformed film; PLA-MC—solution cast film.

## Data Availability

The data presented in this study are available on request from the corresponding author.

## Supplementary Materials

The following are available online at https://www.mdpi.com/article/10.3390/ma14133672/s1, Figure S1: Water contact angles for treated and untreated PLA films.
