# Supplementary figures and images for: Discharge Plasma Treatment as an Efficient Tool for Improved Poly(lactide) Adhesive–Wood Interactions
Source: Materials (Basel). 2021 Jun 30;14(13):3672. doi: 10.3390/ma14133672 (PMC8269815; doi:10.3390/ma14133672)

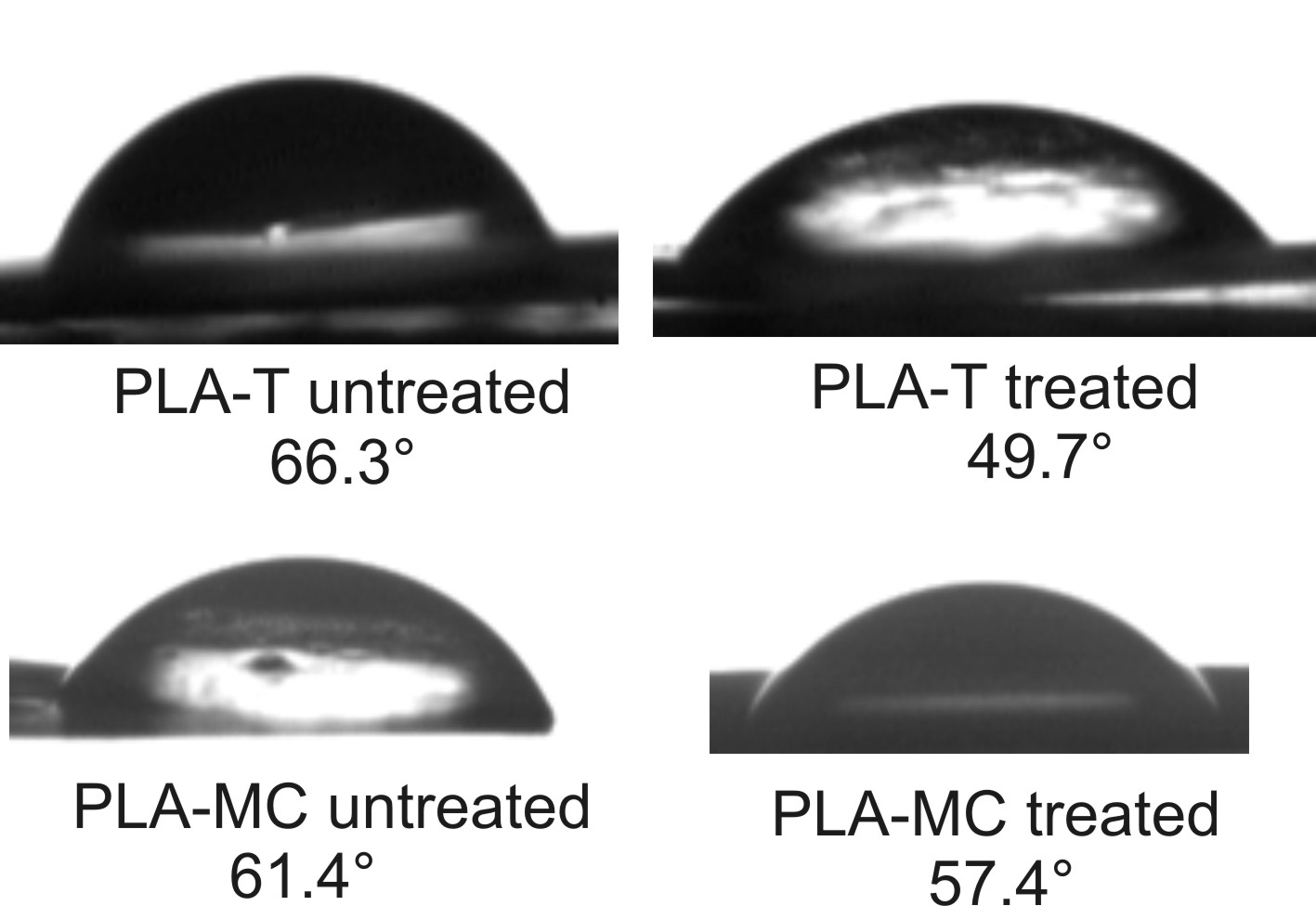

Supplement: Supplementary file 1 [file materials-14-03672-s001.zip › materials-1257409-supplementary.jpg]
